# Rain may improve survival from direct lightning strikes to the human head

**DOI:** 10.1038/s41598-023-50563-w

**Published:** 2024-02-09

**Authors:** René Machts, Alexander Hunold, Christian Drebenstedt, Michael Rock, Carsten Leu, Jens Haueisen

**Affiliations:** 1 Institute of Biomedical Engineering and Informatics, Technische Universität Ilmenau, 98693 Ilmenau, Germany; 2 Group for Lightning and Surge Protection, Technische Universität Ilmenau, 98693 Ilmenau, Germany; 3 Institute of Electrical Engineering, Leipzig University of Applied Sciences, 04251 Leipzig, Germany

**Keywords:** Neuroscience, Natural hazards, Signs and symptoms, Engineering

## Abstract

There is evidence that humans can survive a direct lightning strike to the head. Our question is: could water (rain) on the skin contribute to an increase in the survival rate? We measure the influence of rain during high-energy direct lightning strikes on a realistic three-compartment human head phantom. We find a lower number of perforations and eroded areas near the lightning strike impact points on the head phantom when rain was applied compared to no rain. Current amplitudes in the brain were lower with rain compared to no rain before a fully formed flashover. We conclude that rain on the scalp potentially contributes to the survival rate of 70–90% due to: (1) lower current exposition in the brain before a fully formed flashover, and (2) reduced mechanical and thermal damage.

## Introduction

Lightning can occur during thunderstorms and discharges may have currents of larger than 200 kA in case of cloud-to-ground strikes^1^. Humans are endangered outdoors during a thunderstorm because they can be struck by lightning directly^2–4^. Approximately 5% of all lightning injuries to humans are caused by a direct lightning strike and about 30% by a side flash (remaining injuries are caused by lightning-induced step and touch voltage or upward streamer)^4^. It is noticeable that lightning victims with burns on the head died more often due to cardiac arrest probably because of nervous system complications^4^.

However, the mortality over all the five known mechanisms of how a lightning strike can affect humans is between 10% and 30%^2,3,5^. Uman^6^ has estimated that about 75% of lightning victims suffered cardiopulmonary arrest and the remaining 25% damage to the central nervous system. Despite other influencing factors, it might be assumed that the formation of a surface flashover across the human body is a relevant cause of how people can survive a lightning strike if they are unable to go indoors when thunder roars^6–9^. The surface flashover is defined as a discharge path along the outer skin caused by a high voltage difference between the entry and exit point of current across the body^10^. In the case of a surface flashover (named flashover in the following) the highest fraction of lightning current flows in the flashover channel outside the human body and only a few amperes in the human tissues as shown by theoretical studies and by phantom experiments^4,6–12^.

Previous studies and experiments have not considered the influence of rain on the skin on the formation of flashovers. Cooray^10^ hypothesized in a theoretical study (using a single resistor model to represent the human body) that wet skin e.g. due to rain could reduce the needed voltage to form the flashover and consequently the current exposure of the human body. Ohashi and colleagues^13^ indicated a higher survival rate of animals with wet skin (5 of 10 survived) versus dry skin (3 of 10 survived) in their experiments. However, the effect of water on the skin caused by the rain typically accompanying thunderstorms on the current distributions inside a human, especially the human head, is not known.

Consequently, we aimed to analyze the effects of rain on the skin on the formation of the flashover and the current distribution in human head phantoms.

## Methods

### Creation of human head phantom

We created in total two human head phantoms. Each of them comprised the three main compartments (scalp, skull (neurocranium), and intracranial volume) based on a CT data set^14^. Molds were used to cast each compartment starting with the intracranial volume (brain), followed by the skull, and finally the scalp. The basic casting material was 2% agarose (Agarose Broad Range, Carl Roth GmbH+ Co. KG, Karlsruhe, Germany) in deionized water doped with different amounts of sodium chloride, carbon black or graphite to define the dielectric properties of the compartments in a frequency range from 20 Hz to 1 MHz. An amount of 0.17%NaCl was added to cast the brain compartment resulting in an electrical conductivity of 0.12 S/m to 0.38 S/m and a relative permittivity of 72.5·10^6^ down to 930 in the frequency range from 20 Hz to 1 MHz. An amount of 0.01% NaCl and 4% graphite (Graphite d50, Algin-Chemie e. K., Neustadt-Glewe, Germany) was added to cast the skull compartment resulting in an electrical conductivity of 0.024 S/m to 0.063 S/m and a relative permittivity 9·10^6^ down to 196 in the frequency range from 20 Hz to 1 MHz. An amount of 0.17% NaCl and 4% carbon black (carbon fiber powder, EMV Vega, Recklinghausen, Germany) was added to cast the scalp compartment resulting in an electrical conductivity of 0.054 S/m to 0.63 S/m and a relative permittivity of 43·10^6^ down to 1290 in the frequency range from 20 Hz to 1 MHz. The head phantom casting process and its dimensions are described in^8,9^. The cast head phantom had a moist outer surface due to the usage of agarose as a basic casting material comparable to the moist skin of a human.

### Experimental setup on the current pulse generator

**Figure 1:**
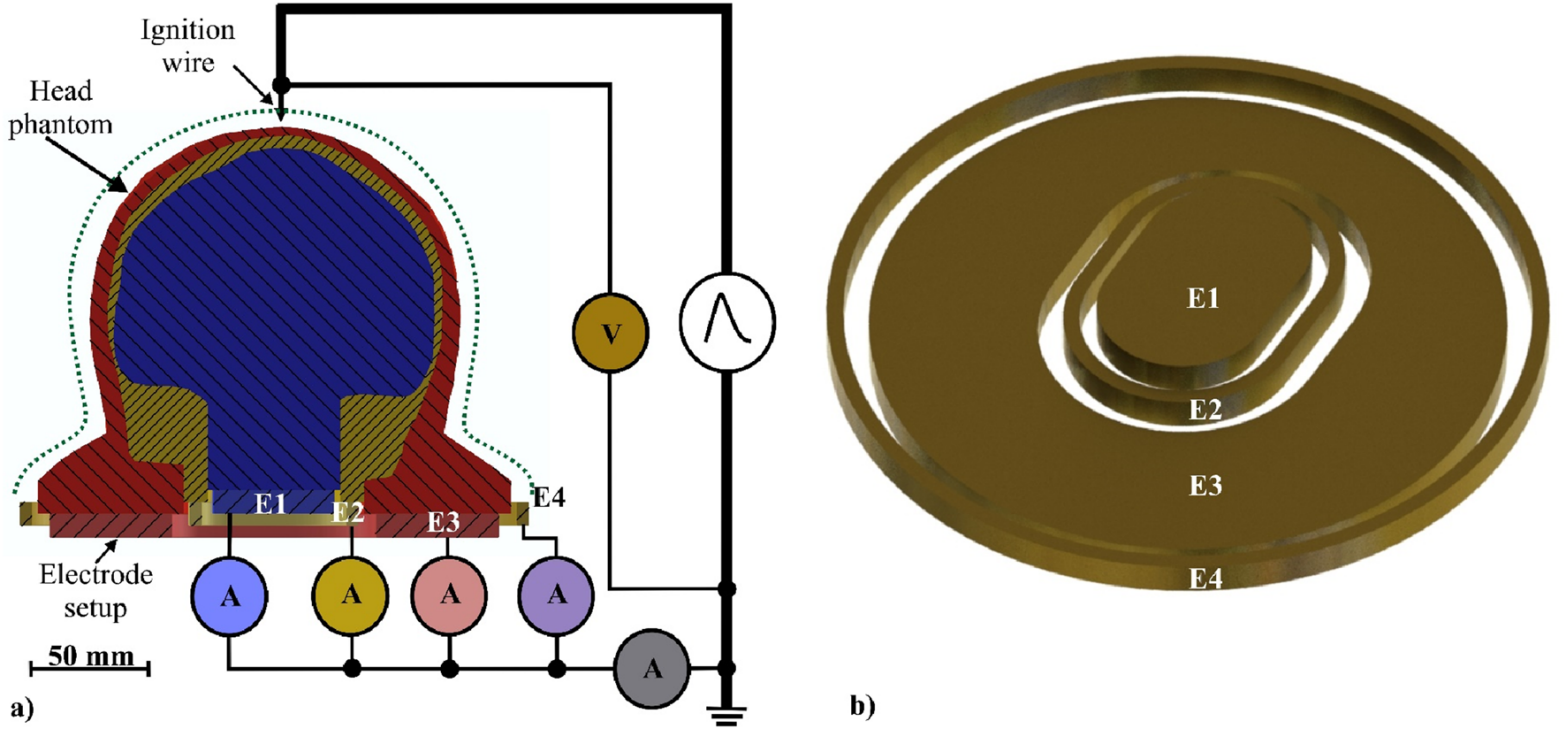
Measurement setup of the head phantom and electrode setup. (**a**) A coronal cut through the complete setup. Each head phantom was placed on such an electrode setup. Electrode E1 contacted the brain (blue), E2 the skull (yellow), E3 the scalp (red), and E4 collected current in case of flashover. Each current of the electrodes E1–E4 and the total current were measured by current monitors (A). The voltage across the head phantom was measured by a high-voltage probe (V). The dotted green line represents simulated rain on the scalp used in the second test series (wet). The current pulses were applied using an ignition wire. (**b**) The 3D rendered geometry of the electrode setup from a side-top view.

We placed each head phantom on an electrode setup consisting of four separate electrodes (Fig. 1)^8^. The three innermost electrodes (E1–E3) contacted the head phantom compartments. The outermost electrode (E4) was used to collect the current in the flashover channel if a discharge propagated over the head phantom to electrode E4. Discharges and flashovers were optically detected by using a single-lens reflex camera (EOS 5D Mark II, Canon Inc., Tokyo, Japan).

Each head phantom including the grounded electrode setup was placed in an examination chamber of a positive 10/350 µs current pulse generator (IP176/12S of HIGHVOLT Prueftechnik Dresden GmbH, Dresden, Germany). The 10/350 µs current pulse generator emulated the high current effects of a direct lightning strike. We used the maximum possible current (42 kA) and voltage (12 kV) of this pulse generator resulting in an applied energy of about 150 kJ for every single discharge. We applied ten discharges on each of the head phantoms. Contrary to the chosen pulse waveform and polarity, about 90% of ground lightning is negative including shorter time parameters^1^. However, the stroke currents for positive lightning are higher and longer, thus with larger specific energy content. Consequently, our setup represented a worst-case approach.

The current pulse generator operated on the principle of discharging a capacitor bank (max. 2.45 mF) in a heavily damped series resonant circuit and delivered aperiodic pulse currents (10/350 µs, non-oscillating) that approximate positive real lightning first stroke currents. The waveform was affected by the high impedance (head phantom) at the output.

We varied the conditions for each head phantom in two test series. In the first test series, the phantom was used without any additions. The first test series is called “dry”. In the second test series, 20 ml of a mixture of 0.0025% sodium chloride in deionized water was homogeneously sprayed on the scalp compartment using a nebulizer before each applied discharge. This mixture resulted in an electrical conductivity of 0.005 S/m and corresponded to the conductivity of rain^15^. This procedure represented rain on the scalp and is called “wet”.

For both test series, an ignition wire (copper, diameter 0.1 mm) was used because of the relatively low voltage of the generator. This wire was placed above the vertex of the head phantom leaving an air gap of 4 mm. We replaced the ignition wire after each applied discharge.

Five current monitors (Pearson Electronics Inc., Palo Alto, CA, United States) captured the current in each electrode (E1–E4) and the total current. The voltage across the head phantoms was measured by a high-voltage probe (PHV 4002-3, PMK GmbH, Kassel, Germany). The measurement setup is shown in Fig. 1.

## Results

### Optically detected flashover

**Figure 2:**
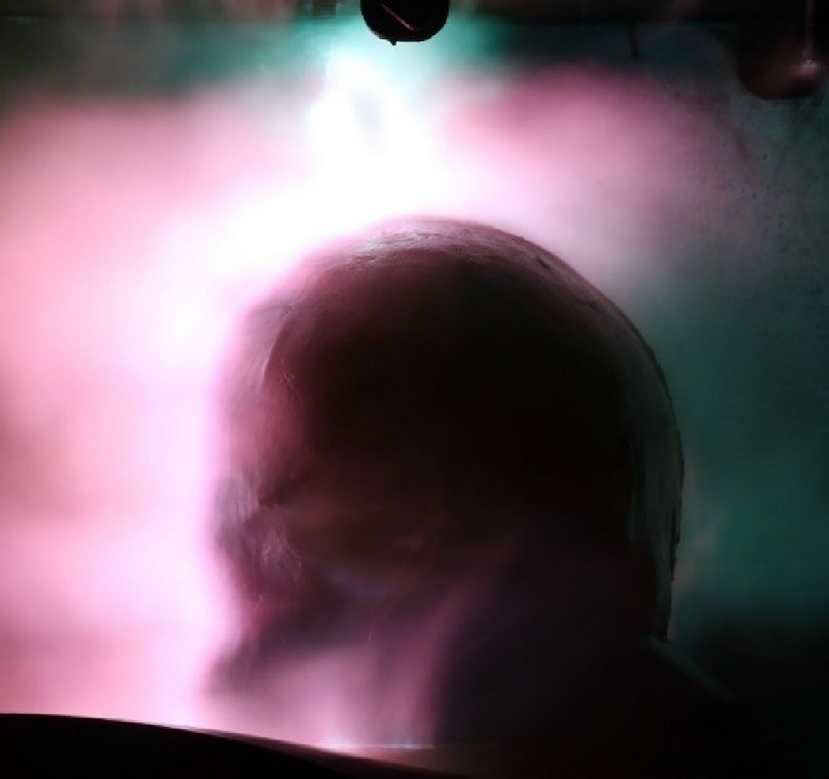
Long-term exposure photography (aperture F/16, exposure time 30 s, focal length 400 mm, ISO-100, neutral density filter 1000) of an observed flashover across the head phantom of the first test series (dry).

We observed a fully formed flashover during each applied current pulse. The flashover always propagated from the output of the current pulse generator over the scalp to electrode E4. A flashover on the head phantom is shown in Fig. 2. The spatial propagation of the flashovers was found to be different for each discharge. Similar optically detected flashovers were observed in^8,9^ across the head phantoms used.

### Voltage and current waveforms

**Figure 3:**
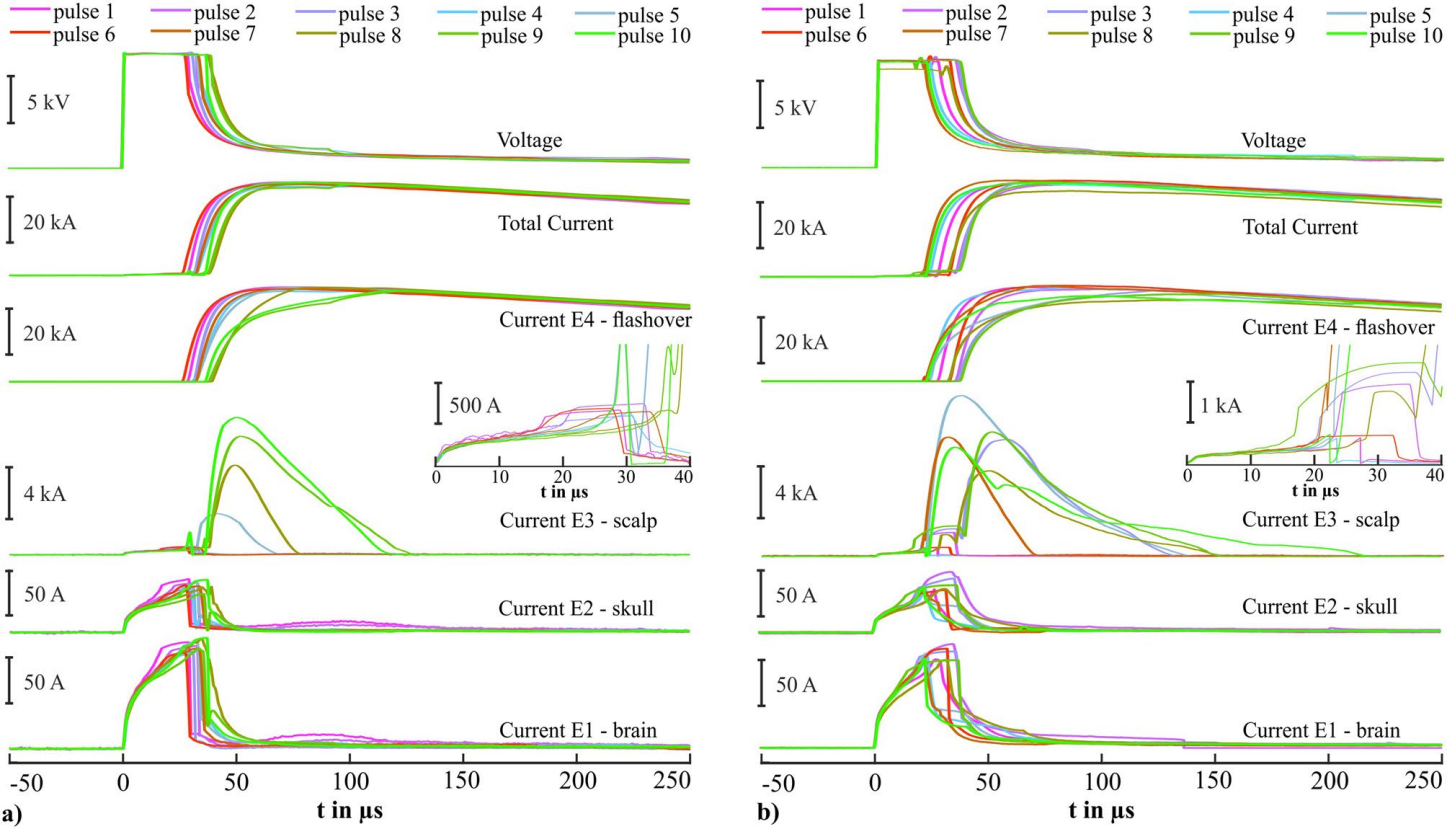
Waveforms (n = 10) of voltage and currents of the first test series (dry) in (**a**) and of the second test series (wet) in (**b**). The inset represents the waveforms of the currents in E3 (scalp) between 0 and 40 µs.

**Figure 4:**
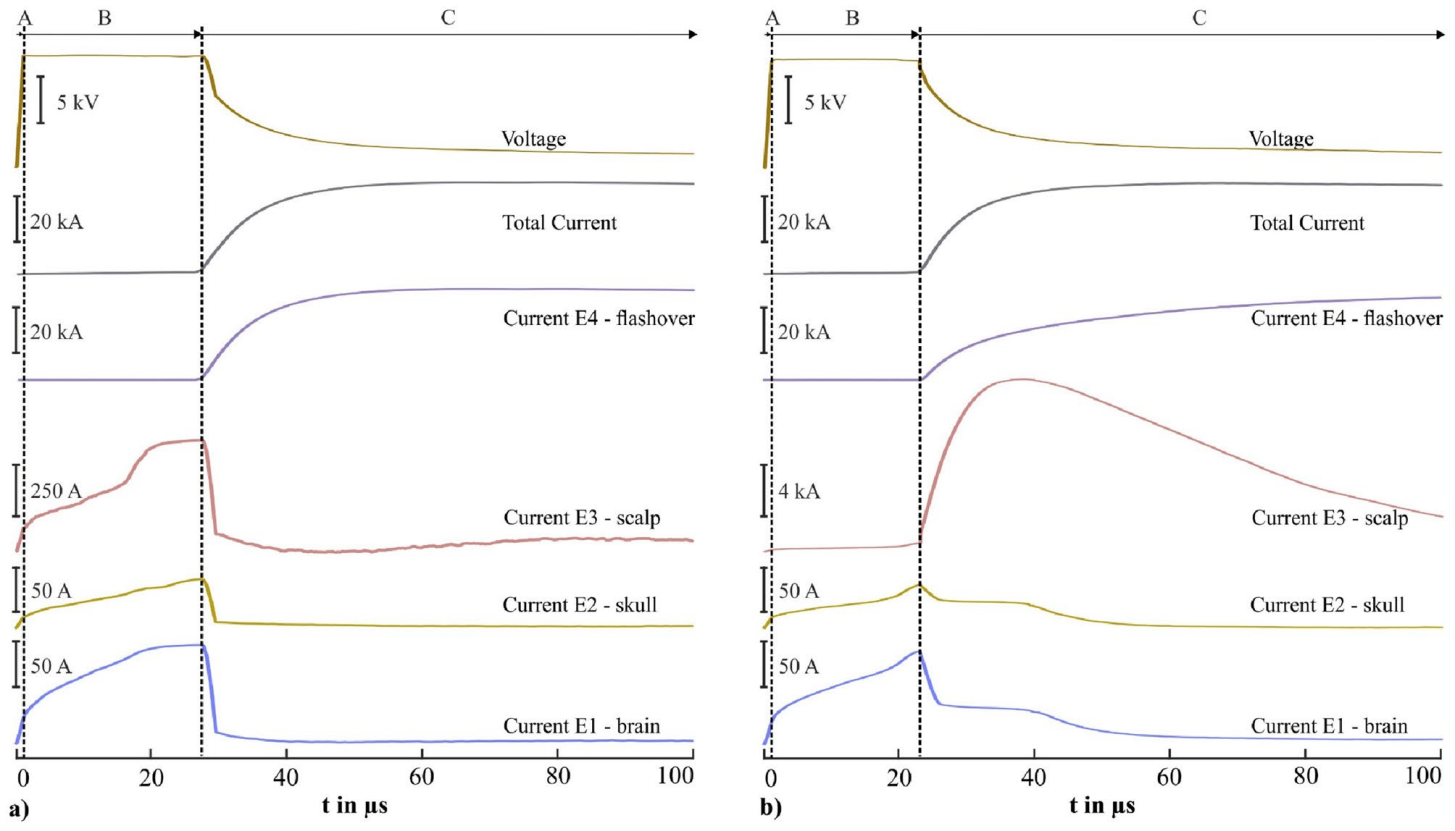
Detailed presentation of waveforms of voltage and currents of the first test series (dry) in (**a**) and second test series (wet) in (**b**) as representative for two cases. Pulse number 6 is shown in (**a**) as an example of no high current in electrode E3 and pulse number 5 is shown in (**b**) as an example of a high current in electrode E3. The color coding of voltage and currents waveforms corresponds to the color coding in Fig. 1a. Three time intervals A, B, and C are shown in both figures. Time interval A starts at 0 µs. Time interval C continues beyond the depicted length of 100 µs and ends with the completed pulse.

Figure 3 depicts the ten recorded waveforms of voltage and current of the first test series without simulated rain (Fig. 3a) and the second test series with simulated rain (Fig. 3b). Qualitatively, we found comparable waveforms in both test series. We observed differences in E3: either no high current pulse in E3 or a high current pulse in E3. Figure 4 shows both cases using a finer time scale and one pulse each as an example.

In the first test series, the voltage started to increase rapidly to an almost constant voltage of about 12 kV within 1.2 µs (SD: 0.03 µs) on average at the time point t = 0 µs (ignition of the current generator). Also, the currents in E1–E3 and the total current began to rise at the first time interval of 0 µs to 1.2 µs on average (marked in Fig. 4 as A). At 1.2 µs on average, the currents in E1–E3 and the total current continued to rise with a reduced rate of increase until 32.6 µs (SD: 2.6 µs) on average. The voltage was almost constant in the second time interval (marked in Fig. 4 as B). After a time of 32.6 µs (SD: 2.6 µs) on average the voltage collapsed (below 2 kV). The currents in E1, E2, and E3 collapsed below a few amperes. The total current and the current in E4 began to rapidly increase after this time, finally reached their maximum, and decreased after their maximum. The identified time point (32.6 µs on average) marked the flashover. No high current pulse was identified after that time point in E3 in six cases (Fig. 4a, as a representative example). The third time interval is marked in Fig. 4 as C.

In four cases (pulses 5, 8, 9, 10) during the third time interval, the current in E3 decreased and then increased to a maximum between 3.7 and 12.2 kA and then decreased again. The current in E4 increased during the decrease of E3 in these four cases (Fig. 4b, as a representative example). The current commutated from E3 to E4.

We identified the three described time intervals in the second test series. The voltage increased at t = 0 µs within 1.2 µs (SD: 0.02 µs) on average to an almost constant voltage of about 12 kV. The currents in E1–E3 and the total current showed qualitatively similar behavior. The currents in E1, E2, and E3 and the total current continued the rise with a reduced rate of increase until 30.5 µs (SD: 6.1 µs) on average. After this time the currents in E1, E2, and E3 as well as the voltage collapsed (below 2 kV). The total current and the current in E4 rapidly increased as previously described in the third time interval (flashover).

In five cases during the third time interval, the current in E3 decreased and then increased to a maximum between 9.1 and 10.4 kA and then decreased again. The current in E4 increased during the decrease of E3 in these five cases. In one case (pulse 5) the current directly increased to a maximum of 13.4 kA without a decrease before. The current in E4 also increased during the decrease of E3 in this case. In the other four cases (pulses 1, 2, 4, and 6), no high currents were observed in E3.

**Figure 5:**
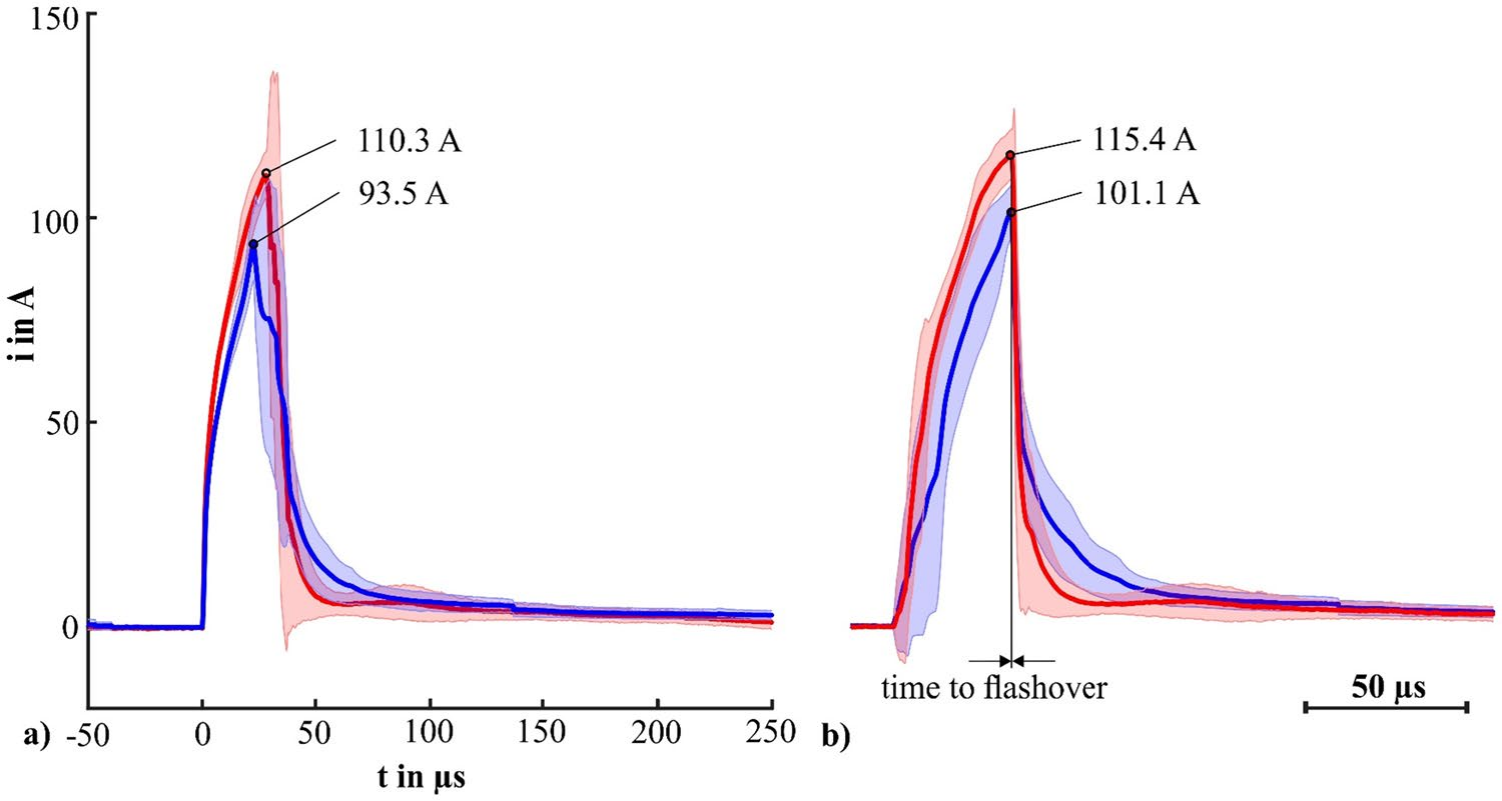
Average current waveforms (n = 10) in E1 (brain) of the first test series (dry) marked red and the second test series (wet) marked blue. Shading indicates standard deviation. (**a**) Identical time alignment as shown in Fig. 3. (**b**) Time alignment at the flashover.

Figure 5 depicts the averaged waveforms (n = 10) of the currents in E1 (brain) of the first (dry) and second (wet) test series in two different time alignments. Figure 5a, shows the identical time alignment of Figs. 3 and 4, where the averaged current of the second (wet) test series reached a lower maximum (93.5 A) in comparison with the first (dry) test series (110.3 A). After the flashover, the average current in the wet test series was higher than the current in the dry test series for a period of a few microseconds. However, the amplitudes are considerably smaller (dry test series 49%-95% smaller, wet test series 42%–93% smaller) for this period in comparison to the maximum. Thereafter, both averaged currents converged to the same amplitude.

Because the flashover is the most significant event during the experiment and has considerable variability in time, we performed a second averaging, where we used the time point of the flashover for the time alignment. Figure 5b, shows the resulting waveforms for the current of both test series. Before the flashover, the rate of increase was higher for the dry test series compared to the wet test series. A maximum average current of 115.4 A was observed in the dry test series and a current of 101.1 A in the wet test series. After the flashover, while both currents decrease the current in the dry test series decreased faster than the one in the wet test series. After about 60 µs both currents converged.

### Current distribution

The relative and absolute current distributions for both test series are shown in Table 1 for the time point immediately before a fully formed flashover. We always found a similar pattern: the highest fraction of current flowed in E3 (scalp) followed by E1 (brain) and E2 (skull). For the phantom with rain, a higher amount of current was observed in the scalp (absolute value approximately doubled compared to dry condition). A significantly (p < 0.05, t-test) lower amount of current flowed in E1 (brain) before flashover for the phantom with rain.

**Table 1:** Relative and absolute current distribution (both averaged over 10 separate discharges) before fully formed flashover (illustrated in Fig. 5, right, for currents in E1) for the test series without (dry) and with rain (wet).

Test series	E3 (scalp) in %	E3 (scalp) in A	E2 (skull) in %	E2 (skull) in A	E1 (brain) in %	E1 (brain) in A
Dry	81.3	733.4 (SD: 395.5)	5.8	52.7 (SD: 4.83)	12.8	115.4 (SD: 5.87)
Wet	90.3	1410.3 (SD: 753.6)	3.2	50.9 (SD: 6.34)	6.4	101.1 (SD: 6.21)

Standard deviations (SD) accompanying each average current estimate are included.

Most of the current (92.0–97.4%) flowed in electrode E4 on the time point of the maximum total current in both test series (Table 2). In these cases, the flashover was fully formed across the head phantoms. The scalp carried more current for the phantom with rain when compared to the phantom without rain.

**Table 2:** Relative and absolute current distribution (both averaged over 10 separate discharges) at maximum total current for the test series without (dry) and with rain (wet).

Test series	E3 (scalp) in %	E3 (scalp) in A	E2 (skull) in %	E2 (skull) in A	E1 (brain) in %	E1 (brain) in A	E4 (flashover) in %	E4 (flashover) in kA
Dry	2.63	1084.3 (SD:2293)	0.008	3.2 (SD:2.85)	0.014	5.8 (SD:3.57)	97.4	40.2 (SD:2.25)
Wet	7.97	3192.9 (SD:3088)	0.008	3.0 (SD:1.51)	0.019	7.5 (SD:2.19)	92.0	36.9 (SD:3.41)

Standard deviations (SD) accompanying each average current estimate are included.

The brain and skull compartments were similarly exposed for both test series. In four respectively in seven cases a pulsed current flowed after the start of flashover in E3 (scalp). This caused the high standard deviation in the scalp compartment.

### Specific energy in the brain compartment

We analyzed the specific energy (action integral) in the brain compartment from 0 to 250 µs for both test series using $W/R=\int {{\text{i}}}^{2}{\text{dt}}$ according to Rakov and Uman^1^. An averaged specific energy of 302 mJ/Ω (SD: 44 mJ/Ω) was found in the test series without rain in the brain compartment and a significantly (p < 0.05, t-test) lower amount of 204 mJ/Ω (SD: 59 mJ/Ω) in the test series with rain.

### Examination of the head phantom

**Figure 6:**
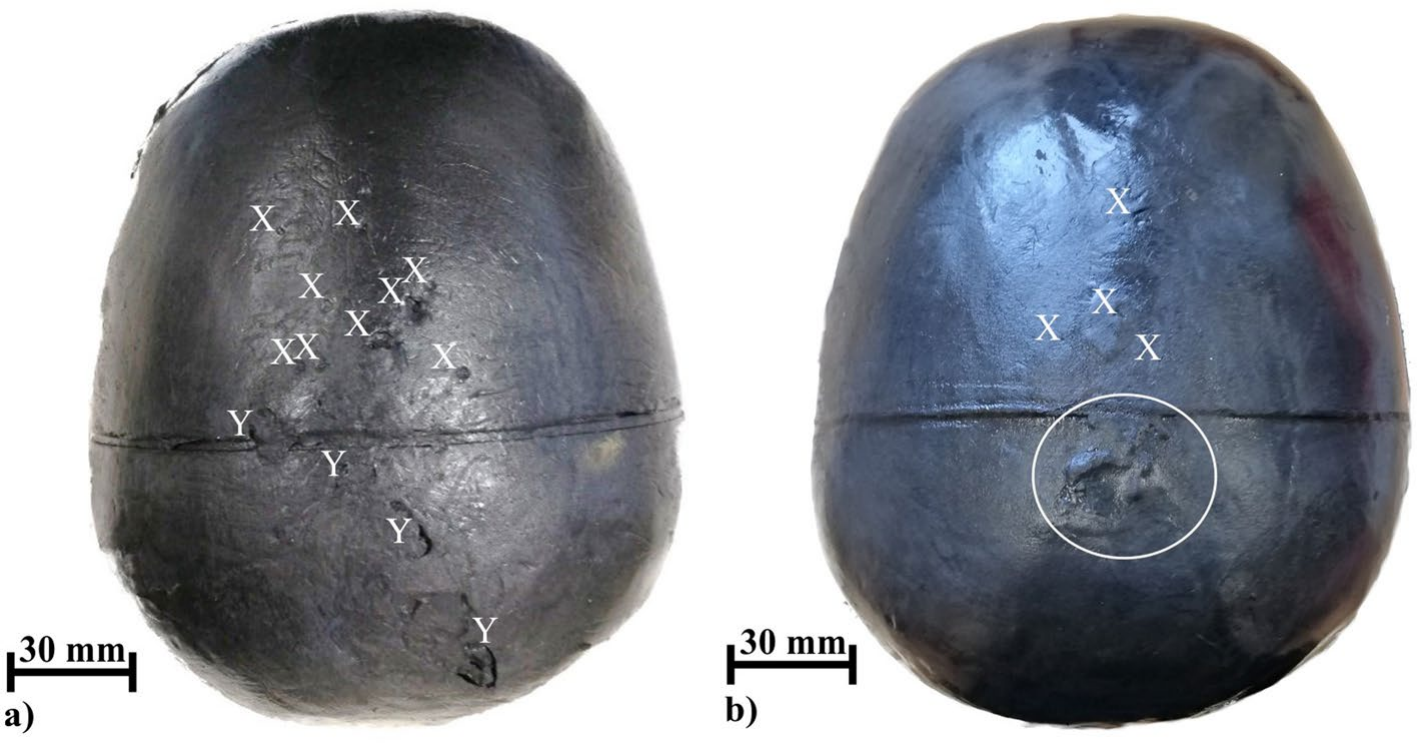
Top view of two examined head phantoms after the first test series without rain in (**a**) and second test series with rain in (**b**). Nine impact points (X) were found on the scalp of the head phantom used in the first test series (dry). Four cracks (Y) were identified. The scalp of this head phantom felt dehydrated. Four smaller impact points below 2 mm (X) were found on the scalp of the head phantom used in the second test series (wet). The scalp felt not dehydrated. The irregular geometry on the scalp marked with an ellipsoid (white) was caused by the casting process.

We visually examined each head phantom after each test series. Qualitatively, the head phantom of the test series without rain was more damaged than the one for the test series with rain (Fig. 6). The phantom of the test series without rain showed more impact points on the scalp and the scalp was more dehydrated (before the test, the phantom surface always feels wet and slippery and after the test, the phantom surface always feels dry and porous) in comparison to the one with rain. For the phantom of the test series with rain, we observed only four small perforations (diameter < 2 mm) of the scalp.

## Discussion

Our experiments on human head phantoms provide practical evidence for the theoretically postulated effect that rain-wet skin might have better lightning strike protective behavior than dry skin^10^. When rain is sprayed on the phantom, we find on average a 12.5% (14.3 A) lower current exposition before a fully formed flashover and on average a 32.5% (98 mJ/Ω) lower specific energy in the brain compartment. These findings could contribute to the explanation of the increased survival rate in the animal experiments (dry skin 3 of 10 survived versus wet skin 5 of 10 survived) of Ohashi et al.^13^. Thermal and mechanical damage caused by plasma during the discharge^1^ were also found to be lower for the head phantom with rain (9 impact points on dry versus 4 impact points and without cracks on the scalp with wet skin).

We speculate that, consequently, burns^4^ will manifest mostly on the scalp and not in underlying compartments^8^. We expect similar effects in case of real lightning strikes into humans during rainfall in thunderstorms. The rain distribution on our phantom is not a fully homogenous water layer (water drops form in a random fashion) which is similar to the situation for humans during rainfall. Consequently, the arc might form differently for each experiment. Moreover, the vaporizing water layer may reduce the temperature on the skin and at the same time, the resulting water vapor slightly blows the arc channel away from the skin^10,16^. To put the effect in perspective, surviving a lightning strike can have many reasons. One such mechanism might be the formation of a flashover, where most of the current commutates from the body compartments to the flashover channel^6,8^. This commutation in current has a much higher impact on survival (over 90% of the current flowing in the flashover channel) compared to the additional effect caused by the rain on the surface of the head.

Some limitations of our study need to be considered. A positive lightning current exceeds in 50% of the cases an amplitude of 35 kA^1^ and in general positive lightning currents most frequently reach amplitudes between 6 and 24 kA^17,18^. The experiments shown here consider probably only up to 50% of lightning current amplitudes that occur in nature.

Uman^6^ described three time intervals during direct lightning strike to a human: 1. upward-connecting leader phase, 2. initial return stroke phase, and 3. surface flashover phase. We can associate the three identified time intervals (A, B, C) of our experiments with these definitions. Time interval A is characterized by a rising time of voltage across the head phantom and an increasing current through the head phantom within about 1.2 µs. Time interval A can be associated to the upward-connecting leader phase. We mention that a formation of a real leader is not possible in our experiments. The ignition wire serves as a leader. Time interval B (initial return stroke phase) is characterized by an almost constant voltage of about 12 kV across the head phantom and a further increasing current through the head phantom within about 30 µs. The surface flashover phase, time interval C in our experiments, is dominated by a high pulse current outside the head phantom (in E4) and a collapsing voltage across the head phantom until the end. The remaining current through the head phantom rapidly commutates in the flashover channel resulting in a collapse from about 115 A down to 5 A in the brain compartment for example.

The constant voltage (about 12 kV) before flashover (time interval B) is caused by the limitation of the current pulse generator in combination with the head phantom. The head phantom has a higher impedance (few kΩ, high capacitive properties) in comparison to a conductor and a lower impedance in comparison to an insulator. Consequently, the impedance of the head phantom results in a voltage across the head phantom and a discharge (low impedance) which is formed at the outer surface after ignition (time interval A). The discharge propagates to the grounded electrodes resulting in an increasing current during the constant voltage (time interval B). If the discharge reaches the grounded electrode (E4) a surface flashover is formed.

Real lightning would produce a pulse-shaped voltage with a much higher amplitude of some MV up to some 10 MV^1,6^. The voltage amplitude and the pulse form predominantly influence the time intervals (A and B) until flashover^19,20^. The limited low maximum charging voltage of 12 kV of our current pulse generator explains the time intervals of several microseconds in comparison to several nanoseconds^10^ characterized for natural lightning accidents. We expect a lower current exposition of the brain compartments during a real lightning strike due to the shorter time intervals in contrast to our head phantom experiments.

We found variability in the measured current magnitudes and current waveforms after flashover in electrode E3 (scalp). In these cases, a discharge between E3 and E4 probably occurred due to mechanical deformation of the head phantoms. The mechanical impact was higher under wet conditions due to the vaporization of water^10,16^. Consequently, we have measured a high current in E3 in seven cases under wet conditions in comparison to four cases under dry conditions. We speculate that the slower decreasing current in the head phantom under wet conditions in time interval C could be caused by the vaporization of the applied rainwater.

We also observed considerable variability in the specific spatial formation of the flashover (location of the fully formed flashover at the head phantom). This variability occurred when using the same or another head phantom under otherwise identical conditions. We speculate that this variability might be related to the repositioning of the ignition wire for each discharge and the uneven surface of the phantoms due to the production process. We expect a similar variability during lightning strikes to a human head because of the variability of human heads with respect to dielectric and geometric properties.

We note that the head phantom neglected hair on the scalp and complex structures like the sulcus/gyrus structure, the CSF space^21^, or the anisotropic conductivity of white matter^22^ as well as portals of entry (sense orifices of the cranium)^23^. We aim to improve the head phantom and consider these elements in future research. Moreover, we did not consider headgear such as hoods or helmets, which needs further investigation. We speculate that headgear might reduce the amount of current flowing in the scalp, which we found to be increased for the wet condition compared to the dry condition. For practical reasons, we used a head phantom without the remaining parts of a human body. Ultimately, a full-body phantom would allow for the investigation of brain-body interactions since mortality after a lightning strike is determined by several factors including respiratory and cardiac ones. Damage of the central nervous system are estimated to account for 25% of the cases while non-central damage account for 75% of the cases^6,23,24^.

While we report here only the physical, non-biological findings, there are biological implications associated with our findings that should be investigated in future work. For example, the coupling of the electromagnetic field to neuronal mass models, which has been employed for weaker therapeutic currents^25^ and can be applied in transcranial magnetic stimulation^26^, might give insights into the brain and brain-body network interactions after a lightning strike. Also, the investigation of possible effects of electroporation^27^ in conjunction with these models deserves further investigation. As lightning is causing many neurological complications^24,28^, and so gaining mechanistic insight into these altered brain functions is highly desirable.

In conclusion, rain might have a protective effect due to the reduction of the brain's temporal and spatial current exposition during a direct strike or side flash in the human head.

## Data Availability

The datasets generated or analyzed during the current study are available in the figshare repository, https://doi.org/10.6084/m9.figshare.22121810.
